# Lymphoplasma Exchange Improves Myasthenia Gravis Exacerbations: A Retrospective Study in a Chinese Center

**DOI:** 10.3389/fimmu.2022.757841

**Published:** 2022-04-14

**Authors:** Song Ouyang, Weifan Yin, Qiuming Zeng, Bijuan Li, Jian Zhang, Weiwei Duan, Yi Li, Yong Liang, Jiaqi Wang, Hong Tan, Huan Yang

**Affiliations:** ^1^ Department of Neurology, Xiangya Hospital, Central South University, Changsha, China; ^2^ Medical Center of Neurology, The First Hospital of Changsha City, South China University, Changsha, China; ^3^ The Second Xiangya Hospital, Central South University, Changsha, China; ^4^ Department of Blood Transfusion, Xiangya Hospital, Central South University, Changsha, China; ^5^ Department of Pathology, University of Iowa, Iowa City, IA, United States

**Keywords:** myasthenia gravis (MG), lymphoplasma exchange (LPE), quantitative myasthenia gravis (QMG), autoimmune diseases, plasma exchange (PE)

## Abstract

**Background:**

Lymphoplasma exchange (LPE), a technique combining plasma exchange with leukapheresis, is emerging as promising treatment for autoimmune diseases. Data on the efficacy and safety of LPE in myasthenia gravis (MG) therapy are scarce. In this study, we aimed to comprehensively review the clinical efficacy, safety, and immunological characteristics of LPE therapy in MG patients.

**Study Design and Methods:**

A Chinese cohort of 276 generalized MG patients in state of exacerbation, including impeding crisis, myasthenia crisis, and preparation for thoracic exsection between January 2014 and December 2020, were evaluated in this study.

**Results:**

A total of 276 patients with a median age of 45.5 ± 16.7 years underwent a total of 635 LPE sessions. Clinical scales of Quantitative Myasthenia Gravis (QMG) scores, Myasthenia Gravis Specific Manual Muscle Testing (MMT) scores, activities of daily living (ADL) scores, and quality of life (QOL) scores were improved during 4 weeks’ follow-up. Adverse effects occurred in 20 out of 276 patients, with 14 patients having one adverse event each. Independent predictive factors for good response to LPE therapy were symptom onset before LPE therapy ≤3 days and age on LPE therapy <50 years of age. LPE decreased the serum levels of antibodies, immunoglobulins, and complements 4 weeks after the first replacement, with decreased levels of interleukin (IL)-17A and interferon (IFN)-γ and increased level of IL-10.

**Conclusion:**

LPE is an effective treatment for MG patients in state of exacerbation and preparation for thymectomy. Early use of LPE on early-onset MG may have good therapeutic effects. The potential mechanism for LPE is the polarization of cytokines from IL-17A, IFN-γ, into IL-10.

## Introduction

Myasthenia gravis (MG) is a chronic neurological autoimmune disease characterized by fluctuating weakness of voluntary muscles. It is a recurrent disease exacerbated by infection, irrational medication, or even harmful emotional stimulation. Most MG patients can maintain remission through treatment with oral glucocorticoids and immunosuppressants in an outpatient setting. However, for patients with impeding crisis or preparing for thymectomy, life-threatening exacerbations such as myasthenia crisis with respiratory fatigue and dysphagia may occur. In an acute exacerbation and in preparation for thoracic surgery, the main goal is rapidly eliminating antibodies so as to reduce damage of the neuromuscular junction. Recently, it has been acknowledged that immunoglobulins, plasma exchange (PE), and immunoadsorption are responsible for a short-term rapid disease remission (1).

The major pathogenic antibodies are anti-acetylcholine receptor (AchR) and anti-muscle-specific kinase (MusK) antibodies, accounting for approximately 80% and 7%–9%, respectively (2). Therapeutic PE has been reported to vigorously remove circulating autoantibodies and immune-competent substances (3), which are attributed to the direct separation of plasma from blood cells with membrane filtration or centrifugation, and reinfusion of the blood with plasma substitutes (4). For decades, PE has shown significant efficacy for acute exacerbation of MG, myasthenia crisis, and preparation for thymectomy in multiple clinical trials (4–6). In the international consensus guidance for MG management, PE has been recommended for impeding crisis and exacerbation of MG prior to the initiation of immunosuppressants (7).

Lymphoplasma exchange (LPE) was developed from traditional PE by combining it with lymphocyte monoculture. It involves removal of part of the patient’s plasma along with activated lymphocytes. LPE is deemed to be superior to PE in that it can not only eliminate inflammatory factors in the periphery but also remove immune-competent cells, such as T and B lymphocytes and platelets (8). Previous studies have shown that LPE therapy is beneficial for rheumatoid arthritis, Guillain–Barre syndrome, and systemic lupus erythematosus (8–10). However, in recent years, only a few studies on LPE therapy for autoimmune diseases have been reported. Our center previously reported that LPE had therapeutic effects on Guillain-Barre syndrome and pustular psoriasis of von Zumbusch (11, 12). Therapeutic LPE has been performed in very few clinical centers, and little is known about the indications and clinical responses to LPE in MG. In this article, we aimed to retrospectively analyze the clinical characteristics, immunological indices, and potential factors of LPE treatment on the outcome of MG exacerbations.

## Materials and Methods

**Figure 1:**
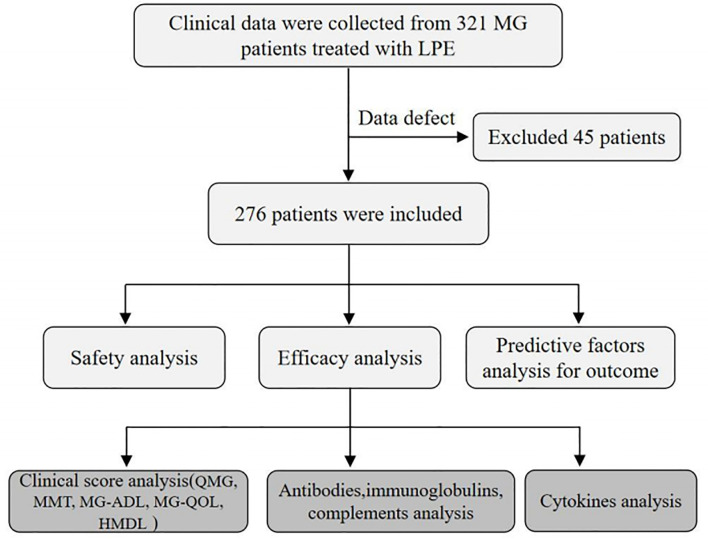
The flowchart designed for this study.

We searched the clinical database of Xiangya Hospital from January 1, 2014, to December 31, 2020, using the terms “MG diagnosis” and “LPE therapy.” We identified 321 potential MG patients and 276 patients satisfied with the following inclusion and exclusion criteria (13–15): (1) MG patients who have complete data on autoantibody tests (anti-AchR, anti-acetylcholinesterase [AchE], anti-ryanodine receptor [RyR], anti-Titin, and anti-MusK), cytokine measurement (IL-4, IL-10, IL-6, IL-17A, IL-23, and interferon [IFN]-γ), and clinical scales of QMG, MMT, ADL, and QOL scores were included in this study; (2) patients included were adults (aged older than 18 years) and in state of impeding or manifesting myasthenic crisis (Quantitative Myasthenia Gravis [QMG] score ≥20, or swallowing plus pulmonary score ≥4); (3) patients with tumors, not including malignant thymoma, were excluded from this analysis; (4) women who were pregnant, lactating, or planning pregnancy were excluded; and (5) patients with renal insufficiency or contraindications for immunoglobulin use were excluded, as it may lead to bias in treatment selection. The design of this study was shown in a flowchart (**Figure 1**). We collected data on the demographic characteristics, clinical features, treatment regimen, and comorbidities of patients included in the medical record system. According to the Myasthenia Gravis Foundation of America (MGFA) classification, the severity of the enrolled MG patients at onset varied from IIIa to V. QMG score improvement more than 3 points after LPE therapy was recognized as “significant improvement.”

For immune index evaluation, we recorded levels of immunoglobulins, cytokines, and complements (C3 and C4) from the clinical laboratory of our hospital at baseline and 1 month after the first replacement of LPE therapy. Concentrations of autoantibodies (anti-AchR, anti-AchE, anti-RyR, anti-Tintin) and cytokine were measured using enzyme-linked immunosorbent assay (ELISA), and levels of anti-MusK antibodies were detected using radioimmunoassay. The levels of immunoglobulins (IgA, IgM, and IgG) and complement (C3, C4) were measured using an automatic biochemical-immune analyzer (MY-B020, China). All participants provided written informed consent.

LPE was performed according to previously reported practices (11, 12, 16). Briefly, we used a COBE Spectra blood cell separator, installed a leukocyte pathway, and collected lymphocytes using density gradient centrifugation and optical colorimetry. For every session, we removed 25–30 ml/kg plasma along with 2–3 × 10^9^ lymphocytes and replaced with equivalent fresh frozen plasma. The treatment regimen was one session a week. 193 patients completed two sessions of LPE treatment, and 83 patients completed three sessions of LPE treatment. All patients were administered prophylactic antiallergic drugs and calcium gluconate.

The studies involving human participants were reviewed and approved by the Medical Ethics Committee of Xiangya Hospital Central South University. We have obtained written informed consent from all study participants. All of the procedures were performed in accordance with the Declaration of Helsinki and relevant policies in China.

### Statistical Analysis

Measurement data are expressed as mean ± standard deviation. Variance analysis was used to compare the mean values of the multiple samples. Student’s t-test was used to compare the mean values of the two independent samples. The Wilcoxon matched-pair signed-rank test was used to assess changes in QMG scores following LPE therapy. Statistical significance was set at p < 0.05. Univariate regression analysis was used to determine the individual variable risk factors for good clinical outcomes of LPE therapy (QMG score ≥ 50% of baseline), and all statistical analyses were performed using SPSS 22.0.

## Results

### Clinical Features of MG Patients With LPE Therapy

We identified 276 patients, including 166 females (60%) and 110 males (40%), who were aged between 18 and 72 years. The MGFA classification of MG patients before LPE therapy varied from classes IIIA to class V. The mean symptom duration was 24.6 ± 20.4 months. Approximately 10.9% of patients had thymic hyperplasia and 11.2% had thymoma. Of these patients, seventy-seven patients had thyroid diseases, such as hyperthyroidism (16.7%) and hypothyroidism (11.2%). During LPE, MG patients were medicated with glucocorticoids (93.5%), immunosuppressants (42.74%), or both (13.9%). The majority of antibodies were anti-AchR (89.8%) and anti-Musk antibodies (6.5%). Details of the baseline clinical characteristics are shown in **Table 1**.

**Table 1:** Demographic baseline characteristics of patients with myasthenia gravis.

Variable	Mean ± SD or n(%)
Sex (female/male)	166 (60)/110 (40)
Ages (years)	45.5 ± 16.7
LPE sessions, times	635
MGFA clinical classification, n (%)	
Class IIIA	85 (30.8)
Class IIIB	70 (25.4)
Class IVA	57 (20.7)
Class IVB	46 (16.7)
Class V	18 (6.5)
Affected muscles, n(%)	
Ocular muscles	207 (75.4)
Laryngopharyngeal muscles	97 (35.1)
Respiratory muscles	78 (28.3)
Limb muscles	199 (72.1)
MG symptom duration, month	23.6 ± 20.4
Thymus hyperplasia	30 (10.9)
Thymoma	31 (11.2)
Thymectomy	46 (16.7)
Hyperthyroidism	32 (11.6)
Hypothyroidism	15 (5.4)
Antibody types, n(%)	
Anti-AchR Ab	248 (89.8)
Anti-AchE Ab	25 (9.1)
Anti-Musk Ab	18 (6.5)
Anti-RyR Ab	21 (7.6)
Anti-Titin Ab	26 (9.4)
Any concomitant medication, n (%)	
Pyridostigmine bromide	260 (94.2)
Steroid	
Prednisone	258 (93.5)
Methylprednisolone sodium succinate	37 (13.4)
Immunosuppressant	117 (42.4)
Azathioprine	30 (10.9)
Tacrolimus	63 (22.8)
Methotrexate	4 (1.4)
Mycophenolate mofetil	20 (7.3)

LPE, lymphoplasma exchange; MG, myasthenia gravis; AchR Ab, acetylcholine receptor antibody; AchE Ab, acetylcholinesterase antibody; Musk Ab, muscle-specific kinase antibody; RyR Ab, ryanodine receptor; SD, standard deviation.

### LPE Improved Clinical Symptoms in Generalized MG Patients

**Figure 2:**
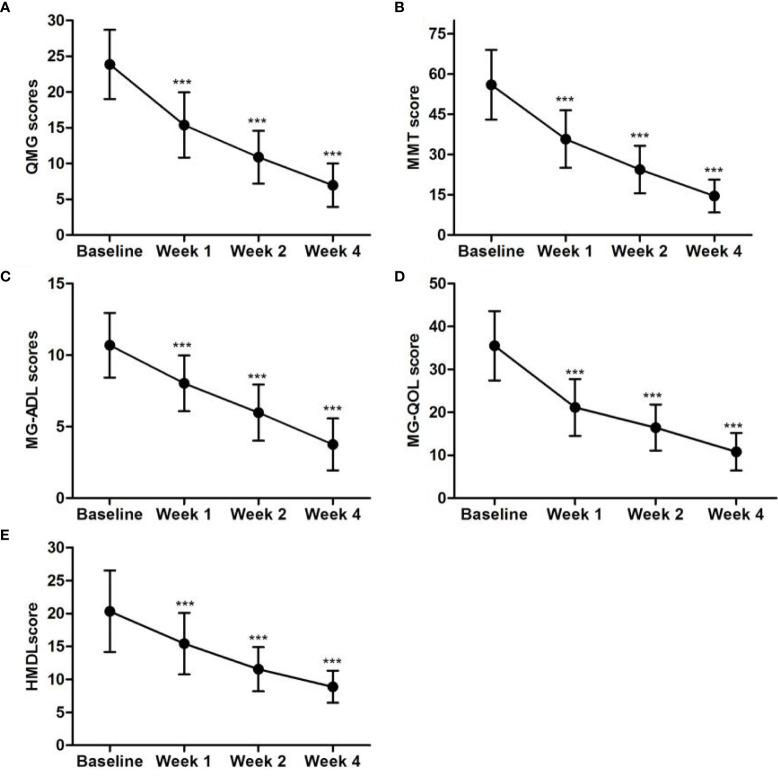
LPE improved clinical symptoms in generalized MG patients. The scores of QMG, MMT, MG-ADL, MG-QOL, and HMDL declined significantly 4 weeks after LPE therapy (n=276, ***p < 0.001). **(A)** QMG scores of MG patients of baseline, week 1, week 2, and week 4 after the first replacement of LPE therapy. **(B)** MMT scores of MG patients of baseline, week 1, week 2, and week 4 after the first replacement of LPE therapy. **(C)** MG-ADL scores of MG patients of baseline, week 1, week 2, and week 4 after the first replacement of LPE therapy. **(D)** MG-QOL scores of MG patients of baseline, week 1, week 2, and week 4 after the first replacement of LPE therapy. **(E)** HMDL scores of MG patients of baseline, week 1, week 2, and week 4 after the first replacement of LPE therapy.Note: LPE, lymphoplasma exchange; MG, myasthenia gravis; QMG, Quantitative Myasthenia Gravis; MMT, Myasthenia Gravis Specific Manual Muscle Testing; MG-ADL, Myasthenia Gravis Specific Activities of Daily Living scale; MG-QOL, myasthenia gravis quality of life; HMDL, Hamilton Depression Scale.

We monitored the scores of clinical scales at baseline, week 1, week 2, and week 4 after the first replacement of LPE therapy for each patient. All patients fulfilled the QMG, MMT, ADL, and QOL scores, while only one hundred patients completed the Hamilton Depression Scale (HMDL). Before LPE therapy, the scores reflecting clinical symptoms were relatively high (QMG, 23.85 ± 4.83; MMT, 56.01 ± 13.02; MG-ADL, 10.69 ± 2.16; MG-QOL, 35.51 ± 8.09; and HMDL, 20.32 ± 26.17). One week after LPE therapy, the scores decreased gradually (QMG, 15.4 ± 4.57; MMT, 35.77 ± 10.75; MG-ADL, 8.10 ± 1.90; MG-QOL, 23.13 ± 6.62; and HMDL, 15.42 ± 4.67). All the scores further reduced 4 weeks after LPE therapy, with QMG, 6.97 ± 3.03; MMT, 14.57 ± 6.13; MG-ADL, 3.70 ± 1.86; MG-QOL, 10.82 ± 4.37; and HMDL, 8.88 ± 2.45. The detailed scores are shown in **Figure 2**.

### Predictive Factors for Outcome of LPE Therapy on MG Patients

In this study, 270 out of 276 patients responded to LPE with an improvement in QMG score ≥3 points. We divided the responsive patients into two groups: one group had a QMG score improvement of more than 50% of baseline and the other had improvement less than 50% of baseline. We studied the clinical factors that might influence the outcome of LPE therapy. Continuous variables, such as the age at onset, symptom duration before LPE therapy, antibody types, and concomitant medication, were converted into categorical variables according to the ratios of patient’s occurrence. Univariate logistic regression showed that age at LPE therapy <50 years (odds ratio [OR] = 2.35, p = 0.018), time between symptom onset and LPE therapy ≤3 days (OR = 3.289, p = 0.049), concomitant medication with glucocorticoids (OR = 1.238, p = 0.05), and advanced stage (stage IIIa to IV) (OR = 2.395, p = 0.041) were significantly associated with clinical improvement. The multivariable logistic regression analysis confirmed the time between symptom onset and LPE therapy ≤3 days (OR = 3.045 [1.231–8.465], p = 0.031) and age at LPE therapy <50 years (OR = 2.045 [0.931–4.465], p = 0.015) as independent predictive factors that significantly affected the clinical improvement of MG (**Table 2**).

**Table 2:** Predictive factors affecting the outcome of LPE therapy on groups with QMG score improvement more than 50 percentage.

Variables	OR	95% CI	p value
Basic data
Female/male	1.325	0.654–2.873	0.873
Age on LPE <50 years	2.351	1.657–3.736	0.018*
Time between onset and LPE ≤3 days	3.289	1.239–3.689	0.049*
Pulmonary disease	3.387	1.895–6.597	0.052
Cardiac disease	2.873	0.367–3.689	0.098
Liver disease	1.783	0.797–4.572	0.257
Diabetes mellitus	0.967	1.237–6.723	0.872
Hypertension	2.016	0.786–5.376	0.572
Smoker	1.863	1.276–2.753	0.237
Alcohol addicted	1.647	1.357–4.751	0.731
≥3 diseases (kidney, cardiac, lung, diabetes, tumors)	0.346	0.886–2.978	0.325
Thymus hyperplasia	1.347	0.785–9.652	0.076
Thymoma	3.257	0.837–12.045	0.085
Thymectomy	4.687	1.351–5.376	0.061
Hypothyroidism	1.763	0.689–4.215	0.137
Hyperthyroidism	2.876	0.884–3.268	0.435
Hepatitis B	0.857	0.659–6.379	0.134
MGFA clinical classification before LPE
Class III+IV	1.238	0.742–2.856	0.047*
Class V	2.689	1.211–5.467	0.693
Antibody types
Anti-AchR Ab	3.576	2.247–3.249	0.091
Anti-AchE Ab	2.346	1.019–5.326	0.653
Anti-Musk Ab	1.785	0.874–2.792	0.059
Anti-RyR Ab	1.937	3.435–8.157	0.713
Anti-Tintin Ab	2.437	1.079–6.534	0.134
Concomitant MG medication, n (%)
Pyridostigmine bromide	2.135	0.667–2.736	0.119
Glucocorticoids	1.238	1.645–3.746	0.045*
Immunosuppressant
Azathioprine	0.768	2.794–6.475	0.376
Tacrolimus	1.347	0.768–3.147	0.136
Methotrexate	2.645	1.741–3.732	0.294
Mycophenolate mofetil	1.967	0.659–2.715	0.356
Stepwise multivariate logistic regression
Time between onset and LPE ≤ days	3.045	1.231–8.465	0.031*
Age on LPE <50 years	2.045	0.931–4.465	0.015*

270 patients were divided into two groups according to QMG scores improvement. One group was those with improvement more than 50% of baseline, and the other group was those with improvement less than 50% of baseline. The table showed odds ratio and 95% confidence intervals of clinical findings associated with different MG groups. Multivariable logistic regression analysis was used to statistically confirm predictive factors with p < 0.05 which showed significance (*p < 0.05).

LPE, lymphoplasma exchange; QMG, quantitative myasthenia gravis; OR, odds ratio; CI, confidence interval; MGFA, Myasthenia Gravis Foundation of America; AchR Ab, acetylcholine receptor antibody; AchE Ab, acetylcholinesterase antibody; Musk Ab, muscle-specific kinase antibody; RyR Ab, ryanodine receptor; MG, myasthenia gravis.

### Effects of LPE on Serum Levels of Antibodies, Immunoglobulins, and Complements

**Figure 3:**
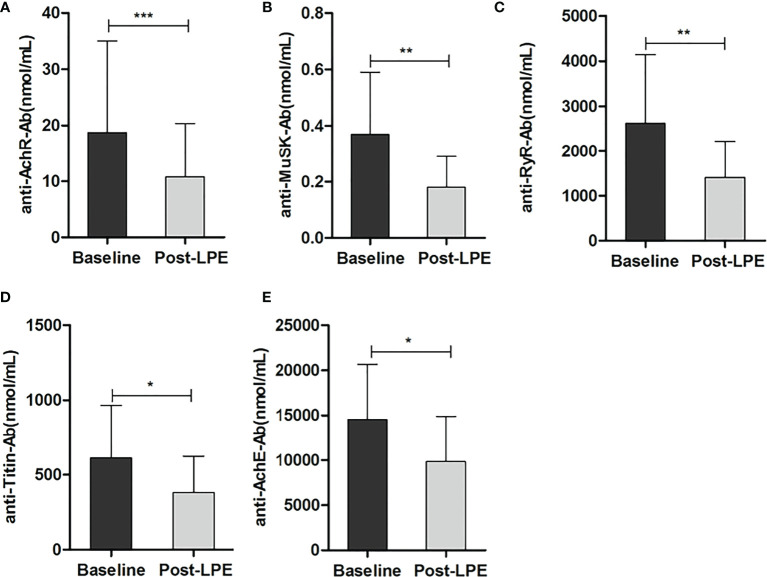
LPE reduced serum levels of autoantibodies after LPE therapy. **(A)** Effect of LPE on the anti-AchR antibody (n = 248). **(B)** Effect of LPE on anti-MusK antibody (n = 18). **(C)** Effect of LPE on the anti-RyR antibody (n = 21). **(D)** Effect of LPE on anti-Titin antibody(n = 26). **(E)** Effect of LPE on anti-AchE antibody (n = 25) (*p < 0.05, **p < 0.01, ***p < 0.001).LPE, lymphoplasma exchange; AchR Ab, acetylcholine receptor antibody; AchE Ab, acetylcholinesterase antibody; Musk Ab, anti-muscle-specific kinase antibody; RyR Ab, anti-ryanodine receptor; MG, myasthenia gravis.

**Figure 4:**
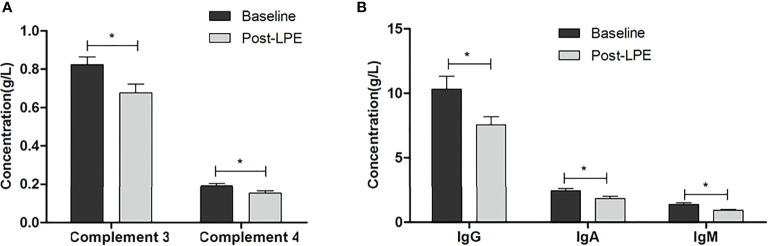
LPE reduced serum levels of immunoglobulins and complements after LPE therapy. **(A)** LPE reduced serum concentrations of complement C3 and C4 (n = 186). **(B)** LPE reduced serum concentrations of immunoglobulins (IgA, IgG, IgM) (n = 166) (*p < 0.05). LPE, lymphoplasma exchange.

Four weeks after the first session of LPE therapy, the levels of the anti-AchR antibody decreased significantly, compared with that before therapy (10.78 ± 9.62 vs. 18.75 ± 16.3 nmol/ml, p < 0.05, n = 248). Levels of anti-AchE, anti-Musk, anti-Ryr, and anti-Titin decreased after LPE therapy (**Figure 3**). Similarly, the levels of immunoglobulin (IgA, IgM, IgG) and complement (C3, C4) were significantly reduced compared with those before therapy (**Figure 4**).

### Effects of LPE on Cytokines in Periphery

**Figure 5:**
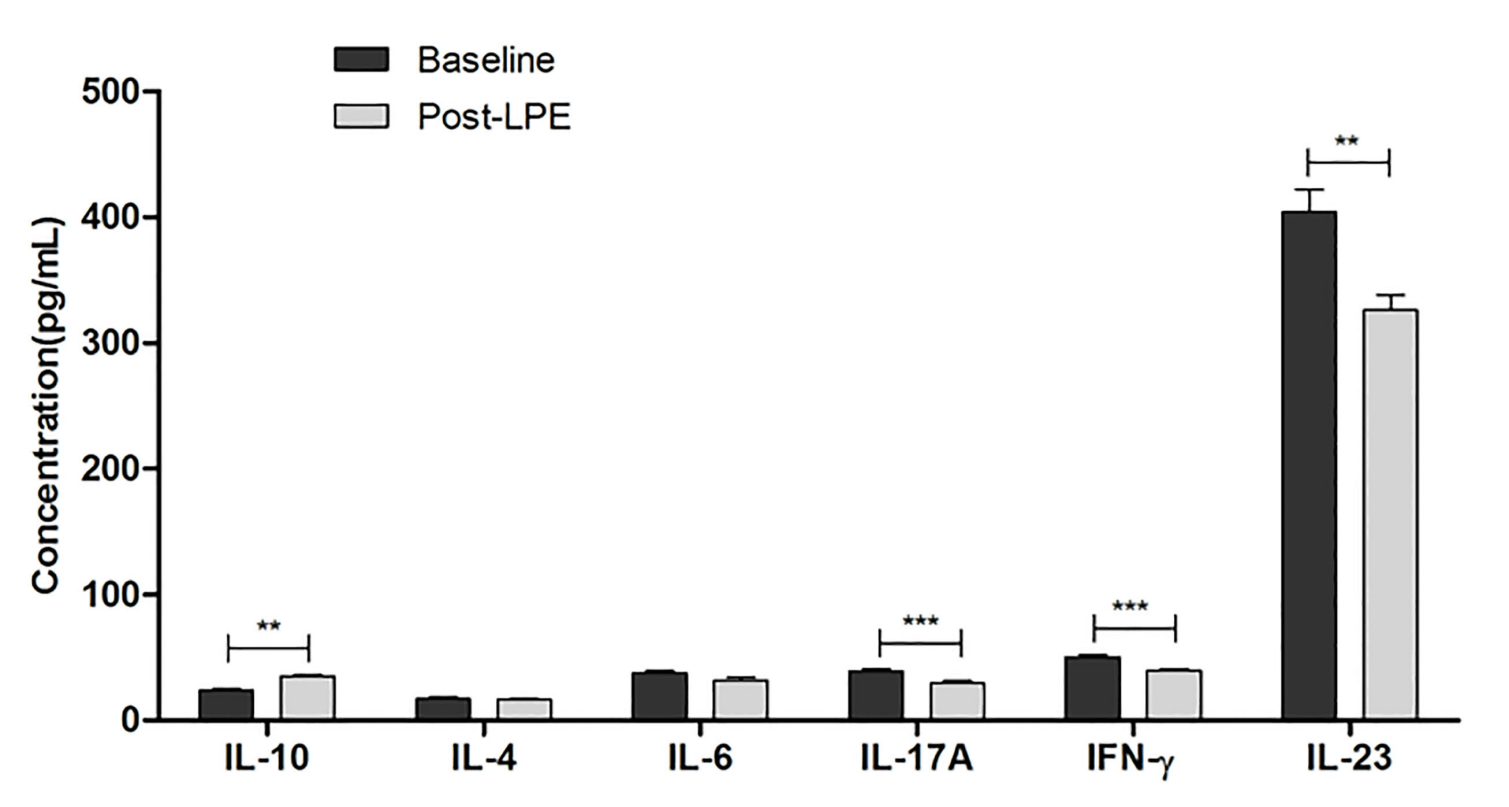
Serum levels of IL-4, IL-10, IL-6, IL-17A, IL-23, and IFN-γ before and after LPE therapy were detected in 87 MG patients. Cytokines were detected with ELISA from peripheral blood of MG patients and healthy controls (**p < 0.01, ***p < 0.001). ELISA, enzyme-linked immunosorbent assay; IL, interleukin; IFN, interferon; LPE, lymphoplasma exchange.

In 87 out of 273 MG patients, serum levels of interleukin (IL)-4, IL-10, IL-6, IL-17A, IL-23, and IFN-γ before and 4 weeks after the first session of LPE therapy were detected by ELISA. IL-17A, IFN-γ, and IL-23 decreased significantly after LPE, while IL-10 increased during therapy, and the levels of IL-4 and IL-6 did not change significantly during the LPE therapy (**Figure 5**).

### Adverse Effects of LPE

In 26 out of 635 single LPE sessions (4.1%), adverse effects occurred in 20 out of 276 patients (14 patients had one adverse event each). None of the patients had an adverse event of related death. The most common adverse effects were allergic reaction and hypocalcemia that occurred in three patients, respectively. After the first replacement of LPE, one patient experienced post-infusion systemic reactions, and one patient had vasovagal syncope. Local adverse events were infection and bleeding (two cases of bleeding and one case of infection due to vascular access, respectively). Emotional adverse effects occurred in two patients, both of which manifested as sleep disorders. Two patients experienced moderate hypotension and recovered after crystalloid infusion. Gastrointestinal symptoms occurred in four patients (one patient had diarrhea, another had nausea and vomiting), who were administered symptomatic treatment. One patient had systematic infection without sepsis, who manifested with fever and cough. This patient had a higher C-reactive protein, procalcitonin, and leukocyte ratio compared with that before LPE therapy. He recovered several days after antibiotic medication. One patient suffered from influenza after the second session of LPE treatment and recovered with symptomatic treatment. Non-specific symptoms (two patients with pain due to vascular access and two patients with headache) were also observed. Details of the adverse effects are shown in **Table 3**.

**Table 3:** Adverse events (AEs) during therapeutic lymphoplasma exchange (n = 635 sessions).

Adverse events	No.	Treatment
Local infection due to vascular access	1	Local antiseptics
Local bleeding due to vascular access	2	Local compression
Post-infusion systemic reactions	1	Symptomatic treatment
Influenza	1	Symptomatic treatment
Nausea and vomiting	1	Metoclopramide hydrochloride
Diarrhea	1	Montmorillonite powder
Headache	2	Loxoprofen sodium tablets
Pain due to vascular access	2	Peripheral analgesics
Electrolyte imbalance (hypocalcemia)	3	10% calcium gluconate
Allergic reaction	3	Antihistamines + prednisolone
Moderate hypotension	2	Crystalloid infusion
Coagulation imbalance	3	Specific substitution
Vasovagal syncope	1	Symptomatic treatment
Systemic infection without sepsis	1	Antibiotics
Sleep disorder	2	Symptomatic treatment
Total adverse events	26	

AE, adverse events.

## Discussion

We analyzed 635 single LPE sessions in 276 individual patients, which represent the largest retrospective cohort on LPE therapy to date. Previously, the investigators noticed encouraging therapeutic effects of LPE on several cases of acute systemic lupus erythematosus, rheumatoid arthritis, and acute renal allograft rejection (9, 10, 17). Recently, Luo et al. reported the therapeutic effects of LPE in only 12 cases of Guillain-Barre syndrome (8). Consistent with earlier reports, we concluded that LPE therapy improved MG exacerbation by recording a sustained clinical improvement with subjective and objective scales at 4 weeks post LPE therapy, which indicated good clinical management and daily activity (18, 19). Our findings of variations in immune indices could supplement the existing literature with new aspects.

In this study, we observed a reduction in complement, autoantibodies, and immunoglobulin levels 4 weeks after LPE therapy. This phenomenon is due to the direct elimination of immune substances and is in line with the findings of previous studies of PE but with minor differences (20). Compared to PE, LPE seems to sustain immune suppression for a longer duration. Liu et al. demonstrated that PE efficiently cleared autoantibodies and intercellular adhesion molecule-1 and evoked Treg cells in late-onset MG in the short term (21). Chien et al. also showed that PE had an excellent short effect, and it could adjust the ratio of T helper cells to T suppressor cells after a single session (22). In 2016, Guptill et al. conducted an integrated study of PE effects on immunoglobulins and IgG isotypes, which showed that AchR antibodies IgG, IgG1, and IgG2 were reduced below the reference range at 3 weeks post PE (23). We deduced that the reason for LPE having a longer immune suppression effect than PE was partly due to the reduction of activated lymphocytes along with plasma, although a detailed comparison between PE and LPE needs further elucidation.

Due to the retrospective nature of this study, we could not screen the T-cell subsets of all patients. Instead, we detected cytokines that may reflect related T helper cells. In the pathogenesis of MG, Th1 cells, which secrete IFN-γ, play a key role in the initiation of experimental autoimmune myasthenia gravis (EAMG) (24, 25). IL-4, a main cytokine secreted by Th2 cells, inhibits the proliferation of Th1 cells and exerts an inhibitory function on Th1 (26). Multiple clinical studies have used the ratio of Th1/Th2 to indicate the immune state during therapy for autoimmune diseases (27). Th17 cells, which secrete IL-17, participate in the progression of MG. Hosseini et al. reported that the increased frequency of Th17 cells was closely correlated with the clinical severity of MG patients (28). Treg play a crucial role in immune homeostasis. The frequency or functional deficiency of Treg may lead to an exacerbation of MG (5). IL-6 plays an important role in balancing Th17 and Treg cells by promoting Th17 and inhibiting Treg differentiation (29, 30). Immune treatment exerts its influence by regulating T helper cells and cytokine IL-10. Zhang et al. reported that PE benefits MG patients through cytokines (IL-2, IL-4, IL-10, and IFN-γ) and Treg (31). In multiple sclerosis, PE decreased IL-6 and increased TGF-β expression in patients (32). Our results of cytokine polarization indicated that LPE decreased proinflammatory cytokines IL-12, IL-17A, IFN-γ, and IL-23 but increased IL-10. Numerous studies have reported that plasma exchange exerts therapeutic effects by altering the polarization of cytokine profiles from Th1, Th2, to Th17 and Treg. IL-10, secreted by Treg, was increased after plasma exchange in multiple autoimmune diseases, such as myasthenia gravis (33), thrombotic thrombocytopenic purpura (34), and multiple sclerosis (35). LPE combines plasma exchange with removal of activated lymphocytes, so it has a similar mechanism with PE on cytokine polarization of IL-10 increase, while extending the time duration for immune tolerance. Our group has previously reported that LPE was effective in treatment of Guillain-Barre syndrome patients by directly removing immunoglobulin, complement, monocytes, and fibrinogen as well as polarizing T helper subsets from Th1, Th17 to Th2 and Treg profiles in the peripheral blood (21), which was consistent with our findings in MG patients. The duration for suppressive effects and the increase of IL-10 in our study might partly be due to the sequential use of glucocorticoids and immunosuppressants for long-term immunotherapy. We depicted a preliminary network of cytokines upon LPE effect. Further mechanisms still need to be elucidated.

Usmani et al. showed that PE had a 96% response rate to MG (36). Comparably, our study showed that the efficiency rate of LPE was 98%. Since both studies were in real-world practice, the reason for the high resolution rate may be the continued usage of glucocorticoids and immunosuppressants. It is important to know which patients will have a good response to LPE. In addition, response to PE was good when it was used early (37). We found that age at LPE therapy <50 years, symptom onset before LPE therapy ≤3 days, concomitant medication with glucocorticoids, and advanced stage were associated with clinical improvement. Symptom onset before LPE therapy ≤3 days and age during LPE therapy <50 years were independent predictive factors for a better response to LPE therapy, which may indicate that young patients may have few cardiopulmonary complications and that the earlier the LPE therapy, the better the outcome.

We documented 26 adverse effects during LPE therapy, including infection, non-specific symptoms, gastrointestinal disorders, coagulation imbalance, and allergic reactions, among others. No patient died secondary to adverse events. These adverse effects had similar events but lower rates than those recorded recently in the PE therapy of the intensive unit (38), which improved with symptomatic treatment. We examined systematic infections by measuring clinical manifestation (fever, cough, expectoration, etc.), blood biomedical indexes (leukocyte counts and ratio in blood routine, erythrocyte sedimentation rate, C-reactive protein, procalcitonin, and blood and urine culture), and radiological examination (chest and abdomen CT). The incidence of infection was relatively lower compared with that of plasma exchange (39). The reason for the relatively low rate of adverse effects may be due to the low frequency of LPE therapy once a week, and a fast benefit could be achieved in the short term for patients with severe symptoms.

Some limitations of our study must be highlighted. First, the observation time was inadequate to explore the long-term effects of LPE therapy. Second, the retrospective nature of this study was a limitation of our analysis. Comparative studies are warranted to provide a horizontal competition between a first-line therapy of PE, immunoglobulins, immune adsorption, etc. Third, our results were not stratified according to the severity of MG or differential autoantibody types.

In conclusion, LPE is an effective treatment for MG patients in state of exacerbation. Early use of LPE on early-onset MG may have good therapeutic effects. The potential mechanism for LPE is the polarization of cytokines IL-17A, IFN-γ, and IL-10. These findings need to be confirmed by randomized controlled trials and discussed in detail in further studies.

## Data Availability Statement

The original contributions presented in the study are included in the article/supplementary material. Further inquiries can be directed to the corresponding authors.

## Ethics Statement

The studies involving human participants were reviewed and approved by the Medical Ethics Committee of Xiangya Hospital Central South University. We have obtained written informed consent from all study participants. All of the procedures were performed in accordance with the Declaration of Helsinki and relevant policies in China.

## Author Contributions

HY designed and funded the project. SO and WY performed, analyzed, and discussed the data, took major part in writing the manuscript, and funded the project. QZ discussed the data and provided financial support. BL performed the plasma exchange. JZ discussed the design as well as the data of the study. WD, YiL, JW, and HT took part in designing the research and discussing the data. YoL partly provided financial support. All authors contributed to the article and approved the submitted version.

## Funding

The project described was supported by grants provided by the Natural Science Foundation of China (81771364 and 81801203), Science and Technology Innovation Guidance Project of Hunan Province (2020SK53009, 2020SK53008), and Changsha Municipal Natural Science Foundation (kq2007037).

## Conflict of Interest

The authors declare that the research was conducted in the absence of any commercial or financial relationships that could be construed as a potential conflict of interest.

## Publisher’s Note

All claims expressed in this article are solely those of the authors and do not necessarily represent those of their affiliated organizations, or those of the publisher, the editors and the reviewers. Any product that may be evaluated in this article, or claim that may be made by its manufacturer, is not guaranteed or endorsed by the publisher.
